# Small RNA Plays Important Roles in Virus–Host Interactions

**DOI:** 10.3390/v12111271

**Published:** 2020-11-07

**Authors:** Hui Dai, Weifeng Gu

**Affiliations:** Department of Molecular, Cell and Systems Biology, University of California, Riverside 900 University Avenue, Riverside, CA 92521, USA; huid@ucr.edu

**Keywords:** virus–host interaction, non-coding small RNAs, antivirus, RNAi, RNA phosphatase

## Abstract

Non-coding small RNAs play important roles in virus–host interactions. For hosts, small RNAs can serve as sensors in antiviral pathways including RNAi and CRISPR; for viruses, small RNAs can be involved in viral transcription and replication. This paper covers several recent discoveries on small RNA mediated virus–host interactions, and focuses on influenza virus cap-snatching and a few important virus sensors including PIR-1, RIG-I like protein DRH-1 and piRNAs. The paper also discusses recent advances in mammalian antiviral RNAi.

## 1. Introduction

The study of host–virus interaction is critical for developing effective antivirus strategies and cures. Its importance is fully exemplified in the current emergency of Coronavirus disease 2019 (COVID-19). Viruses interact with hosts using diverse mechanisms across infection stages. A typical mechanism involves protein–protein or protein–lipid interaction when viruses search target cells, such as in the cases of severe acute respiratory syndrome coronavirus 2 (SARS-CoV-2) and influenza virus infections [1,2,3,4]. Once entering cells, viruses hijack host transcription and translation machinery using viral suppressors including proteins and nucleic acids. In addition to protein factors, small RNAs play important roles in virus–host interactions. For example, recent studies demonstrate that influenza A virus (IAV) utilizes host capped non-coding small RNAs as primers to initiate viral mRNA synthesis [5,6]; host microRNAs (miRNA) can be invovled in regulating viral replication, transcription, and translation [7,8]; viral miRNAs can inhibit host antiviral mechanisms [8,9,10]; small interfering RNAs (siRNA), Piwi-interacting RNAs (piRNA), small nuclear RNAs (snRNAs), and CRISPR (clustered regularly interspaced short palindromic repeats) RNAs (crRNA) are all involved in virus–host interaction (Table 1).

Host cells have developed multiple layers of antiviral mechanisms in the endless battles against viruses throughout evolution. In mammals, cellular immunity and humoral immunity based on T, B and other immune cells play critical roles. Host immunity can also be classified as innate and adaptive immunity. Innate immunity constitutes the first layer of immune responses and utilizes physical, chemical, cellular and molecular mechanisms to clear invading viruses while adaptive immunity involves the development of antibody and long term memory primarily involving T and B immune cells. In innate immunity, the interferon (IFN) I, II and III signaling pathways play critical roles in defending host cells against viruses. In these pathways, short double-stranded RNAs (dsRNA) are recognized by the Retinoic acid-inducible gene I (RIG-I) family proteins and stimulate the expression of interferons and downstream factors to clear viruses [22,23].

The recent discoveries in RNA interference (RNAi) and CRISPR demonstrate a novel but long awaited mechanism to clear viruses, i.e., using small RNA sensors to recognize viral nucleic acids and using enzymes to destroy them [21,24]. In RNAi, dsRNAs are processed into 20 to 30-nt siRNAs by Dicer, an RNase III like enzyme [25,26]. Some small RNAs, such as piRNAs and crRNAs, are generated in Dicer-independent manners [21,27,28,29,30,31]. Regardless, small RNAs are primarily used as sensors to monitor target RNAs. Unlike anybody-based target recognition, which involves three-dimensional structures, small RNA-based target recognition only requires primary sequence (one-dimensional), greatly simplifying sensor design. This beauty and simplicity allow quick adaptation of these discoveries to bio-engineering tools for manipulating nucleic acids in all kingdoms of life.

Here, we discuss recent advances in host–virus interactions via small RNAs, primarily focusing on IAV cap-snatching, virus sensors, and mammallian antiviral small RNAs.

## 2. Capped Small RNAs Play Important Roles in IAV mRNA Synthesis

**Figure 1:**
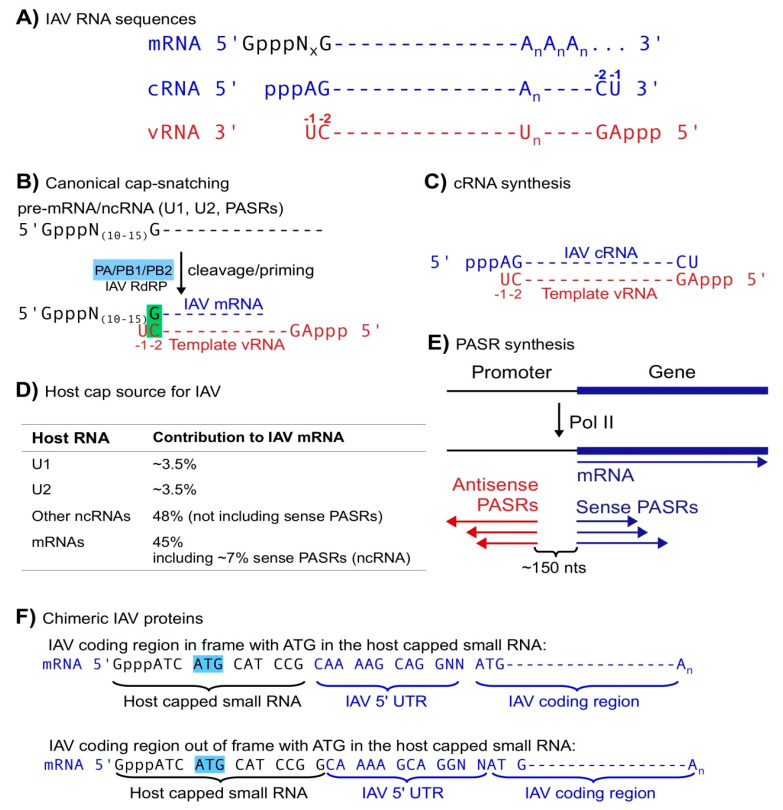
Canonical cap-snatching. (**A**) IAV mRNA, cRNA and vRNA sequences (An represents a poly(A) tail and GpppNx represents a host capped small RNA with x equal to 10 to 15 nts). (**B**) the canonical cap-snatching mechanism: IAV RdRP cleaves host capped RNAs at positions 10–15 nts downstream of the 5′ caps and utilizes the last nt (G) of the resulting host capped small RNAs to anneal with the penultimate (-2, C) nt of template vRNAs, initiating mRNA synthesis. (**C**) Synthesis of IAV cRNA starts with the last nt (-1 U) of template vRNA in a primer-independent manner. (**D**) Host cap sources of IAV mRNAs. (**E**) PASR biogenesis: sense PASRs start at the same transcription start sites as the annotated ”gene” and antisense PASRs are mapped ~150 nts upstream of sense PASRs but on the opposite strand. (**F**) Schemes of chimeric IAV proteins utilizing a start codon ATG in the host capped small RNAs.

The IAV genome is composed of eight negative-sense viral RNAs (vRNA) for generating mRNAs encoding polymerase basic protein 1 (PB1), polymerase basic protein 2 (PB2), polymerase acidic protein (PA), hemagglutinin (HA), nucleoprotein (NP), neuraminidase (NA), matrix protein (M including M1 and M2), and nonstructural protein (NS including NS1 and NS2). PA, PB1 and PB2 constitute the IAV RNA-dependent RNA polymerase (RdRP) complex responsible for RNA transcription and replication; glycoprotein HA and NA on the IAV virion surface are responsible for target cell interaction (virion entry to and release from host cells, respectively); M plays roles in virion assembly and budding; NP binds/protects vRNAs in the virion; NS1 inhibits host RNAi and interferon-mediated innate immunity, and NS2, also called nuclear export protein (NEP), is involved in IAV virion export from host cell nuclei [32,33,34]. In the early stage of infection, IAV RdRP utilizes template vRNAs to generate positive-sense mRNAs, each of which contains a coding frame flanked by a 5′ and 3′ non-coding regions (NCR). Interestingly, a poly(A) tail is added using a stuttering mechanism, which repeatedly utilizes a short poly(U) sequence on template vRNAs to add/extend the poly(A) tail on IAV mRNAs (Figure 1A). Since the poly(U) sequences are not at the 5′ end of template vRNAs, IAV mRNAs are shorter than vRNAs if the poly(A) tail is not counted [32,33,34]. In the late stage of infection, IAV utilizes RdRP to generate complementary RNA (cRNA) using template vRNA and vRNA using template cRNA. vRNA and cRNA are exactly reverse complementary and both bear 5′ triphosphate (ppp) without a poly(A) tail (Figure 1A) [32,33,34].

To make viral proteins, most viruses hijack host translation systems, which usually utilize capped RNAs, i.e., mRNAs, as substrates. To make capped viral mRNAs, viruses can utilize host capping enzymes, which reside in nuclei. However, many RNA viruses have to encode their own RNA capping enzymes since they perform transcription only in cytoplasm, which lacks host RNA capping enzymes [35]. Influenza virus utilizes a special process, cap-snatching, to generate viral mRNAs composed of a host capped small RNA and a virus-encoded RNA [36,37,38]. To generate such mRNAs, the IAV RdRP utilizes PB2 to bind host capped RNAs, PA to cleave at positions 10–15 nucleotides (nt) downstream of the 5′ cap, the last nt, usually G, of the resulting capped small RNAs to anneal with the penultimate nt (−2; always C) of template vRNAs, and the polymerase activity to synthesize viral mRNAs based on vRNA templates (Figure 1A,B; [5,6,11,12,39,40,41,42,43]). Thus, IAV utilizes cap-snatching to obtain caps for its mRNAs from host capped RNAs, indirectly utilizing host capping enzymes. In cap-snatching, host capped small RNAs may appear to serve as primers [6,12]. However, if the last nt (G) of the host capped small RNA is treated as an nt 5′ modified with a capped RNA oligo (Figure 1B), this single-basepair-mediated priming mechanism is actually equivalent to that in de novo RNA synthesis without any primer, the initiation mode utilized by most RNA polymerases including for IAV complementary RNA (cRNA) synthesis (Figure 1C). In summary, cap-snatching allows IAV to obtain caps while maintaining the authenticity of viral RNA sequences.

Host non-coding (nc) small RNAs likely serve as the major cap donors in IAV cap-snatching. For decades, host ncRNAs have not been within the radar range of cap-snatching studies. Based on limited sequencing data and annotations, host mRNAs were initially identified as the major cap source [36,37,38]. However, the short sequences of host capped small RNAs on IAV mRNAs basically allow almost all of them to match host mRNA sequences even if matching was restricted to mRNA transcription start site regions, since most mRNAs utilize multiple transcription start sites. Given that mature mRNAs are not localized in nuclei where cap-snatching occurs, pre-mRNAs were proposed as the authentic donors despite lack of sequencing evidence such as intron-containing sequences [36,37,38]. Several groups have utilized high-throughput sequencing to analyze the cap donor profile of IAV cap-snatching [6,11,12]. Among them, Koppstein et al. and Gu et al. identified U1 and U2 snRNAs as the top cap donors while Sikora et al. did not include snRNAs in their search despite the existence of such sequences (Figure 1D) [6,11,12]. Although mature snRNAs are localized in nuclei, Koppstein et al. speculated that only pre-snRNAs, which share the same sequences with mature snRNAs but bear a 7-methyl Guanosine (m^7^G) cap instead of a 2,2,7 trimethyl Guanosine (m^2,2,7^G) cap (mature snRNAs), are the authentic donors. Their speculation was based on two previous observations: (1) the m^2,2,7^G cap on mRNA has a lower affinity to translation factor eIF4E than m^7^G cap; and (2) the ratio of U1 snRNA cap to U2 cap on IAV mRNAs corresponds well with the transcription rate (representing pre-RNA levels) of U1 and U2 snRNAs rather than the steady-state levels (primarily representing mature RNA levels) [44,45,46]. These arguments may bear flaws since (1) IAV mRNA translation may not require eIF4E [47]; (2) m^2,2,7^G caps may help virus-specific translation such as in human immunodeficiency virus (HIV) translation [48]; and (3) the paper did not examine the U1 and U2 snRNA levels in the IAV-infected cells but only used those in the non-infected cells published previously to correlate pre-snRNA levels to cap usage [45,46]. Actually, Gu et al. demonstrated that U2 contributed a similar number rather than 3-folds of caps as U1 at two post-infection time points [6,12]. This discrepancy can be caused by different experimental designs including cell lines used, infection stages, cloning methods, etc. Regardless, further experiments, for example, an analysis of viral caps using m^2,2,7^G immunoprecipitation, can address whether cap-snatching utilizes mature U1 and U2 snRNAs or only pre-U1 and U2.

Cap-snatching may prefer host capped ncRNAs as substrates. Unlike the other two groups, which only sequenced viral capped RNAs, Gu et al. simultaneously obtained host and viral capped RNAs, allowing them to obtain unique matches to host capped RNAs (substrate) for the capped small RNA parts on IAV mRNAs (product) in the same samples and to obtain cap-snatching rates (product/(product + substrate)). U1/U2 snRNAs combined provided ~7% caps on IAV mRNAs; all known ncRNAs including U1/U2, other snRNAs and snoRNAs provided at least 55% caps; pre-mRNAs provided less than 45% including ~7% snatched from sense promoter-associated small RNAs (PASR), a class of small ncRNAs associated with Pol II transcription initiation (Figure 1D,E; see below) [6]. Although host ncRNAs have a higher snatching rate than mRNAs, Gu et al. did not distinguish pre-RNAs from mature RNAs due to the limitations of their experimental strategy. A transcription rate analysis using global run-on sequencing (GRO-seq) and other methods may answer whether pre-ncRNAs are preferred over pre-mRNAs as cap-snatching substrates [49,50].

PASRs, another type of capped small ncRNAs usually with sizes of less than 200 nts, serve as a significant cap source for IAV mRNAs [6]. Transcription initiation by Pol II usually generates sense PASRs starting exactly at the transcription start sites of annotated mRNAs and other RNAs transcribed by Pol II, and antisense PASRs mapped ~150 nts upstream on the antisense strands (relative to annotated genes; Figure 1E) [49,51,52,53,54,55]. In other words, transcription initiation by Pol II is usually bidirectional or divergent and may pause or fail, generating PASRs, while elongation is usually unidirectional. PASRs serve as piRNA precursors in *C. elegans* and can be processed into miRNAs in animals [13,14]. In theory, sense PASRs bear the same sequences as annotated host mRNAs and other Pol II products. Therefore, the 45% caps on IAV mRNAs thought to be derived from host pre-mRNAs could represent an alternative source, i.e., sense PASRs. Gu et al. found that ~7% IAV mRNA caps were explicitly derived from antisense PASRs, which were mapped to genomic regions without any known annotations [6]. Based on PASR symmetry, they also proposed that sense PASRs may also contribute a similar amount. Therefore, among the 45% IAV mRNA caps assigned to host pre-mRNAs, at least 7% can be traced to sense PASRs, further reducing the contribution of host pre-mRNAs as an IAV mRNA cap source. Othmar et al. showed that IAV RdRP interacts with host Pol II via Pol II C terminal domain (CTD) [56], thereby likely regulating Pol II initiation and elongation. Since PASRs are likely generated by abortive Pol II transcription, it is tempting to propose that IAV RdRP pause Pol II, thus promoting the biogenesis of PASRs for cap-snatching while inhibiting host mRNA elongation, a double jeopardy game to promote virus infection and inhibit host transcription. Further studies are needed to examine this hypothesis.

The chimeric feature of IAV mRNAs allows IAV to generate chimeric proteins. Yuin Ho et al. recently demonstrated that IAV mRNAs can utilize ATG in host capped small RNAs to initiate translation, generating two types of novel proteins containing a few amino acids encoded by host capped small RNAs and the IAV 5′ untranslated region (UTR) or NCRs: one attached to in-framed IAV-encoded proteins and the other attached to out-of-frame IAV-coded “novel proteins” (Figure 1F). Although both types of proteins are expressed at very low levels in the IAV infected cells, they contributed to virulence and were able to initiate host immune response via T cells [57].

**Figure 2:**
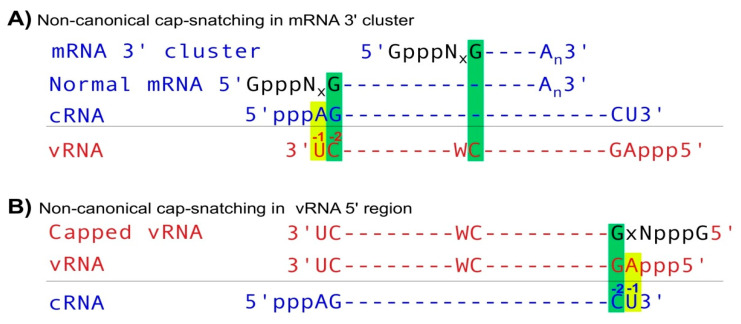
Noncanonical cap-snatching. (**A**) In mRNA 3′ clusters, IAV mRNA transcription starts using the basepairing of between the -1 nt (G) of host capped small RNAs and an internal nt C embedded in a 5′CW (W represents A or U) sequence on template vRNAs. (**B**) In vRNA 5′ regions, IAV RdRP utilizes the -1 nt (G) of host capped small RNAs to anneal with the -2 C of template cRNAs, initiating capped vRNA synthesis.

Li and Hui et al. recently reported that IAV utilizes non-canonical cap-snatching to diversify its mRNAs and ncRNAs [5]. Canonical IAV mRNA synthesis starts using the basepairing of the last nt (usually G) of snatched capped small RNAs and the penultimate (-2) nt (always C) of template vRNAs via cap-snatching (Figure 1B). However, non-canonical cap-snatching occurs primarily in two types of regions. In the first type, named as mRNA 3′ clusters, IAV mRNA synthesis utilizes the basepairing of the last nt (G) of snatched capped small RNAs and an internal C nt on template vRNAs, generating mRNAs or ncRNAs usually covering the last ~300 (up to 1000) nts of normal IAV mRNAs (Figure 2A). In the second type, named as vRNA 5′ regions (Figure 2B), cap-snatching primarily occurs at the second position of IAV vRNA, i.e., using the basepairing of the last nt (G) of snatched capped small RNAs and the -2 nt (C) of template cRNAs to synthesize capped vRNAs (normal vRNAs contain a 5′ triphosphate group and start at the first position, which corresponds to -1 nt of template cRNA). This constitutes a perfect symmetric transcription pattern in which IAV mRNAs primarily start using the template vRNA -2 nt and non-capped cRNAs usually utilize the −1 nt, while capped vRNAs start using the template cRNA -2 nt and non-capped vRNAs predominantly utilize -1 (Figure 2A,B). However, the transcription (capped RNA)/replication (non-capped RNA) activities are different since IAV mRNAs (capped) are expressed at much higher levels than non-capped cRNAs, while the opposite, i.e., much more non-capped RNAs, occurs on vRNA strands. Regardless, like canonical cap-snatching, non-canonical cap-snatching also generates host-tagged (a few amino acids) IAV proteins, host-tagged novel proteins, and many ncRNAs. Although most IAV mRNAs (~98%) are generated using the canonical initiation sites (-2 C of template vRNAs), ~9% NA mRNAs utilize the non-canonical sites (mRNA 3′ cluster). Since NA protein plays critical roles in defining the antigenicity of IAV and is used as the major drug target, it is tempting to assume that these non-canonical cap-snatching events could lead to novel NA proteins, which may contribute to IAV virulence, initiate host immune response and affect drug efficacy. 

## 3. Pattern Recognition Receptors in Antiviral RNAi

Pattern recognition receptors (PRR) play important roles in host immunity against microbial pathogens and viruses. PRRs specifically recognize/bind shared pathogen structures and molecules, collectively called pathogen-associated molecular patterns (PAMP) [58]. In other words, PAMPs serve as pathogen-derived ligands which are recognized and bound by PRRs. Toll-like receptors (TLR) are a type of PRRs that are expressed in immune cells and non-immune cells including epithelial and endothelial cells [59,60]. TLRs recognize/bind microbial pathogens and viruses, initiating a cascade of immune and cellular events including cytokine/interferon production and cell proliferation or apoptosis [59,60]. PAMPs recognized by TLRs include bacterial lipoproteins and peptidoglycans, fungus proteins, small synthetic chemicals, and viral proteins. Several TLRs including TLR3, 7, 8, 9 and 13 can recognize viral nucleic acids including dsRNAs and single-stranded RNAs (ssRNA), viral DNA and bacterial ribosomal RNA (rRNA) sequences [59,60].

RIG-I, another type of PRRs, serves as a virus RNA sensor that recognizes several RNA viruses in mammalian cells and stimulates IFN pathways to clear viruses [61,62]. RIG-I contains a triphosphate binding motif and an ATP-dependent DExD/H box RNA helicase domain, both of which play important roles in recognizing triphosphorylated short viral dsRNA intermediates [22,61,62]. How the RNA helicase domain functions (is unwinding required?) in RIG-I-mediated dsRNA binding remains unexplored. Other RIG-I related genes including melanoma differentiation-associated gene 5 (MDA5) and laboratory of genetics and physiology 2 (LGP2) gene also play roles in antiviral innate immunity [61,63].

*C. elegans* Dicer related helicase 1 (DRH-1) is homologous to mammalian RIG-I. However, DRH-1 only contains an RNA helicase domain while lacking a triphosphate binding domain [64]. Although DRH-1 interacts with Dicer, which is responsible for processing long dsRNAs into primary siRNAs with sizes of 23 nts, it is dispensable for the normal RNAi pathways triggered by long dsRNAs [15,65]. However, DRH-1 is required for antiviral RNAi against a natural *C. elegans* virus, the Orsay virus [66,67]. DRH-1 was initially proposed as a viral dsRNA sensor based on its homology to mammalian RIG-I and its requirement for the biogenesis of virus-derived primary siRNAs via Dicer [66]. However, a recent study showed that DRH-1 is required for the translocation or processivity of Dicer along dsRNA substrates since primary siRNAs were still made but restricted to the terminal regions of viral dsRNAs when DRH-1 is depleted [68]. Whether these terminal siRNAs are loaded to Argonaute or just exist in duplex forms as direct Dicer products remains unexplored. Since Dicer possesses its own DExD/H box RNA helicase domain, the requirement of another helicase partner (DRH-1) in the Dicer complex becomes an intriguing and important question in studying the antiviral mechanism. It is also interesting to dissect the mechanism difference between normal RNAi and antiviral RNAi in *C. elegans*, since DRH-1 is only required for antiviral RNAi.

## 4. Phosphatase Interacting with RNA/RNP 1 (PIR-1) Is Likely Involved in Antiviral RNAi and Serves as a Triphosphate Sensor

**Figure 3:**
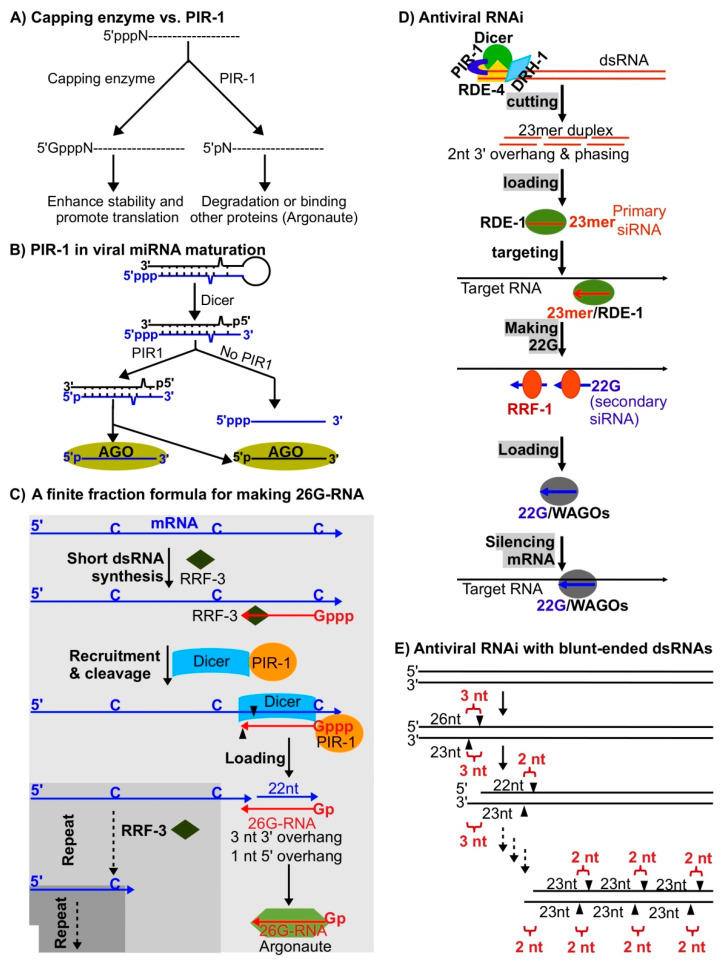
Antiviral RNAi mechanisms. (**A**) PIR-1 and capping enzyme mediated regulations of ppp-RNAs. (**B**) Mammallian PIR1 in viral miRNA maturation. Precursor viral miRNAs transcribed by RNA Pol III are cleaved by Dicer to generate duplex siRNAs, which are processed by PIR1 to convert the 5′ triphosphate (blue) to 5′ monophosphate and then loaded to Argonautes (AGO). Without PIR1, only the 3′ part siRNAs (black) are loaded to AGO. (**C**) 26G-RNA biogenesis: RRF-3 synthesizes short dsRNAs using C nts on template mRNAs, PIR-1/Dicer is recruited to the triphosphorylated dsRNAs, Dicer utilizes a special mode to cleave dsRNAs to generate duplex siRNAs composed of 26G-RNAs and 22mer RNAs with a 3-nt 3′ overhang, PIR-1 removes two phosphates from the triphosphate group on 26G-RNAs, 26G-RNAs are loaded to Argonautes, and the truncated template mRNAs are reused to generate more 26G-RNAs again and again using the same steps as above (the overall process is like a mathematical finite fraction formula). (**D**) Antiviral RNAi mechanism: the Dicer/DRH-1/PIR-1/RDE-1 complex cleaves dsRNAs to generate duplex 23mer primary siRNAs with a 2-nt 3′ overhang, single stranded primary siRNAs are selectively loaded to Argonaute RDE-1 to form primary RNA-induced silencing complexes (RISC), these RISCs bind target RNAs, RdRP RRF-1 is recruited to generate secondary siRNAs (22G-RNAs), 22G-RNAs are loaded to worm-specific Argonautes (WAGO) to form secondary RISCs, and these RISCs silence target RNAs. (**E**) Processing of dsRNAs with blunt ends: the first cut by Dicer generates a duplex siRNA composed of one 26mer and one 23mer siRNAs with a 3-nt overhang, the second cut generates a duplex siRNA composed of one 23mer and one 22mer siRNAs with a 2-nt overhang, and the following processive cuts generate duplex siRNAs composed of two 23mer siRNAs with a 2-nt overhang.

Unlike its paralogs, including the triphosphatase domains of RNA capping enzymes, PIR-1 as an RNA polyphosphatase removes both the β and γ phosphates rather than just the γ phosphate of triphosphorylated RNAs (ppp-RNA) (Figure 3A) [69,70,71,72,73,74,75]. As a result, PIR-1-treated RNAs are usually destined for destruction or further processing instead of for protection/translation as in mRNA capping [76,77]. Mammalian PIR1 (DUSP11) is required for the maturation of viral miRNAs via promoting loading to Argonautes (Figure 3B) and for downregulation of a couple of cellular ncRNAs including vault and Alu RNAs [76,77]. Both the miRNAs and ncRNAs are generated by RNA Pol III, thereby bearing a 5′ triphosphate group and requiring dephosphorylation in the maturation processes. It is likely that these roles may not represent all the functions of mammalian PIR1 since the studies were only based on cell cultures. Further studies at organismal and developmental levels may disclose more important functions of PIR1 in mammalian cells.

*C. elegans* PIR-1 interacts with Dicer [78] and is required for the biogenesis of 26G-RNAs (26G) [74], which regulate thousands of genes in spermatogenesis and embryogenesis [79,80,81,82]. In this biogenesis pathway, PIR-1 recognizes triphosphorylated short dsRNAs generated by worm RNA dependent RNA polymerase (RdRP) RRF-3, recruits Dicer for dsRNA cleavage (generating 26G-RNA precursors) and promotes the loading of 26G-RNAs (26 nts long and preferentially starting with G) to Argonautes by dephosphorylating the triphosphorylated 26G-RNA precursors (Figure 3C) [74]. These observations are consistent with the functions of mammalian PIR1 on the maturation of viral miRNAs [76,77]. Interestingly, here the PIR-1 and Dicer complex utilizes a non-processive but phased manner (finite fraction) to generate 26G-RNAs [74,83]. Technically, this biogenesis manner is equivalent to those used in normal antiviral RNAi pathways, which generate phased siRNAs (23mer siRNAs) processively from long dsRNAs (Figure 3D) (see below) [66].

Our unpublished data suggests that PIR-1 and Dicer function together in *C. elegans* RNAi pathways to silence Orsay virus, a naturally occurring RNA virus originally discovered in a wild isolate of *C. elegans* and thereafter used as a virus model [67]. In *C. elegans*, RNA interference and other small RNA pathways play critical roles in silencing viruses [65,66,68,84]. *C. elegans* serves as a perfect model system to study antiviral small RNAs since it lacks other antiviral pathways including cellular and humoral immunity, interferon pathways, etc. Both viral transgenes and natural viruses have been used to investigate the antiviral RNAi mechanisms [65,66,68,84]. In essence, the antiviral pathway shares the same mechanism as the normal RNAi pathway triggered by any dsRNA introduced either endogenously or exogenously. dsRNAs are recognized by a protein complex consisting of DCR-1 (Dicer), DRH-1, RNAi defective 1 (RDE-1) (Argonaute), and RNAi defective 4 (RDE-4) (dsRNA binding protein) (Figure 3D). DCR-1 cleaves dsRNAs processively to generate phased 23mer siRNAs with 2-nt 3′ overhangs, also called primary siRNAs. In vitro studies have shown that Dicer cannot generate phased 23mer siRNAs with 2-nt 3′ overhangs from blunt-ends or ends bearing a 5′ overhang of dsRNAs [85,86]. When starting from blunt ends, Dicer first cuts dsRNAs, generating a 23mer/26mer duplex siRNA, which bears a 3-nt 3′ overhang, and a truncated dsRNA product also bearing a 3-nt 3′ overhang; Dicer can potentially utilize this truncated dsRNAs to generate phased 23mer siRNAs with a 2-nt 3′ overhang processively (Figure 3E). This initiation step of 23mer/26mer biogenesis was only observed in in vitro assays and has not been explored in small-RNA mediated antiviral pathways. As in other pathways, PIR-1 may be required for the loading of primary siRNAs to Argonaute RDE-1 [76]. Interestingly, primary siRNAs cannot directly silence Orsay virus and have to mediate the antiviral role by binding Argonuate RDE-1 to promote the biogenesis of secondary siRNAs, 22G-RNAs (another triphosphorylated 22-nt small RNAs preferentially starting with G) (Figure 3D) [66,87,88]. The 22G-RNAs serve as an amplified silencing signal to directly silence the targets via unknown mechanisms.

In conclusion, PIR-1 may serve as a sensor of ppp-dsRNAs including viral transcription/replication intermediates and dsRNA intermediates in the 26G-RNA pathway. Interestingly, both types of intermediates are likely short dsRNAs instead of long dsRNAs synthesized using full-sized template RNAs. In antiviral pathways, these dsRNAs may be “abnormal” RdRP products including abortive transcription and erroneous initiation. However, in the 26G-RNA pathway, it is apparent that the mechanism is designed on purpose by the cells.

## 5. piRNAs Serve as Virus Sensors

**Figure 4:**
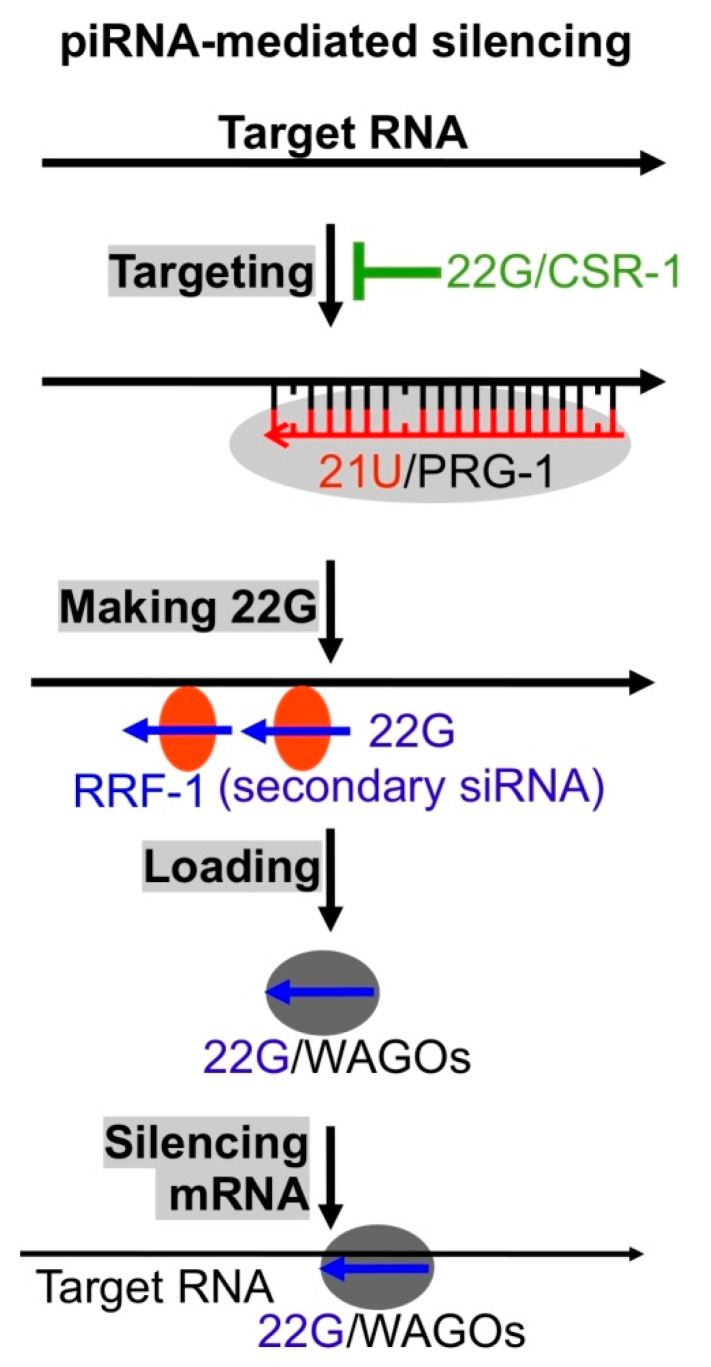
The antivirus sensor piRNA. C. *elegans* 21U-RNA (21U, piRNA)/Argonaute Piwi related gene 1 (PRG-1) complex binds target RNAs with up to 3 mismatches, RdRP RRF-1 is recruited to generate secondary siRNAs (22Gs) using target RNAs as templates, 22Gs are loaded to WAGOs to form secondary RISCs, and these RISCs directly silence target RNAs.

In animal germlines, piRNA, a class of 20 to 30 nts long ncRNAs play critical roles in silencing transposons, maintaining germline genome integrity [27,28,29,31]. Piwi Argonautes utilize piRNAs to recognize target RNAs including viral RNAs and cleave them. Interestingly, most piRNAs (21U-RNAs with size 21 nts and preferentially starting with U) in *C. elegans* do not target transposons. Instead, at least some of them target endogenous RNAs via basepairing with mismatches (Figure 4) [18,19,89,90,91,92]. It is assumed that the ~30,000 piRNAs constitute a repertoire of sensors that recognize any foreign RNAs including viral RNAs via imperfect basepairing (Figure 4) [13,18,89]. Since the relaxed basepairing mechanism may cause self-targeting, many mRNAs expressed in *C. elegans* germline cells are protected via a CSR-1 (Argonaute)/22G-RNA mediated mechanism [87]. This piRNA-targeting/CSR-1 protection mechanism has been tested using germline transgenes [93]. However, since there is no natural virus that can infect *C. elegans* germline cells (Orsay virus only infects intestine cells), the model has not been tested with any live viruses. It is also interesting to see whether the piRNAs of low abundance can protect hosts from viruses since there is no evidence suggesting that the expression of those piRNAs can be induced in response to foreign RNA invasion.

## 6. RNAi Plays Antiviral Roles in Mammalian Cells

IFN (interferon) signaling has been established as a textbook model for clearing viruses in mammalian cells and has withstood scrutiny for several decades. In these pathways, RIG-I related proteins and other virus sensors detect invading viruses and then induce the expression of IFN. IFN engages IFN receptors to activate signal transducer and transcription activator 1 and 2 (STAT1/STAT2), which promote the expression of IFN-stimulated genes (ISG) [9,94]. Among these genes, several factors, including 2′-5′-oligoadenylate synthetase (OAS), protein kinase R (PKR) and RNase L, have been established as important antiviral proteins for degrading invading viruses and shutting down general translation [9,94]. In addition, RIG-I and RIG-I like receptors (RLRs) can negatively regulate RNAi related processes. For example, LGP2, an RLR, represses Dicer-dependent processing of long dsRNAs in mammalian RNAi [95] and miRNA biogenesis by binding trans-activation-responsive region (TAR)-RNA binding protein (TRBP) to compete for its dsRNA-binding sites [96]; RIG-I represses short hairpin RNA induced RNAi by type-I interferon [97].

**Figure 5:**
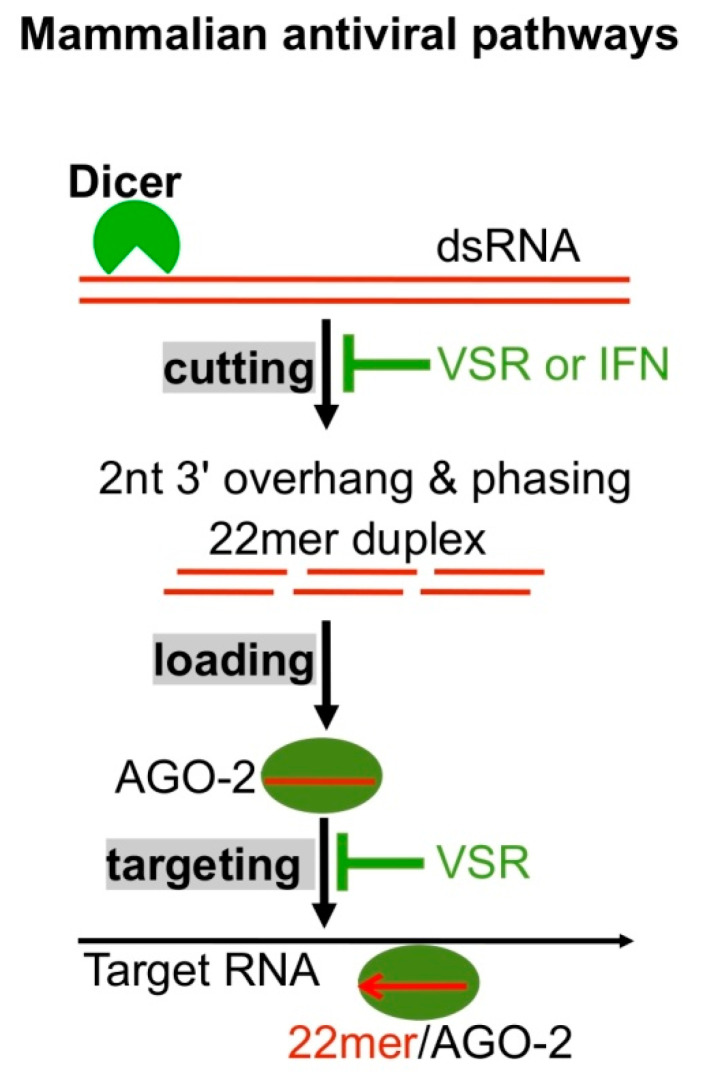
Mammalian antiviral RNAi. Dicer cleaves dsRNAs to generate duplex 22mer siRNAs with a 2-nt 3′ overhang, 22mer siRNAs are loaded to Argonaute AGO2 to form RISCs, and RISCs cleave target RNAs. VSR and IFN signaling suppress Dicer activity; VSR also suppresses RISC activity.

The discovery of virus-derived siRNAs (vsiRNA) in mammalian cells by Mallard et al. and Li et al. not only further expands the functions of RNAi but also challenges the canonical view on mammalian antivirus [98,99], i.e., IFN signaling plays predominant roles in innate immunity. These vsiRNAs bear a typical signature of Dicer-mediated cleavage, i.e., ~22 nts long with a 2-nt 3′ overhang and their biogenesis is dependent on Dicer (Figure 5) [98,99]. Several subsequent studies have confirmed the existence of vsiRNAs in undifferentiated and differentiated cells infected with positive and negative-strand RNA viruses [100,101,102,103]. The discovery of vsiRNAs in differentiated cells is interesting since unlike undifferentiated cells, these cells are usually capable of inducing potent IFN signaling, which inhibits Dicer activity for processing dsRNAs into siRNAs, as demonstrated using artificial long dRNAs (Figure 5) [16,99].

RNA viruses often encode proteins antagonizing host immunity. Many viruses including Nodamura virus (NoV), influenza virus, and Dengue virus-2 utilizes viral suppressors of RNAi (VSR), protein B2, NS1 and 2A respectively, to inhibit Dicer activity (Figure 5) [98,99,102,103]. In consequence, it is difficult to observe vsiRNAs when cells are infected with these wild type viruses. Consistent with this, several experiments exhibited obvious vsiRNAs only in mammalian cells infected with VSR-compromised viruses [98,99,102,103]. In addition, VSR may inhibit IFN signaling [10,104,105]. The VSR-mediated dual inhibitions complicate the previous discoveries on vsiRNA biogenesis since the inhibition of Dicer leads to less vsiRNAs and that of IFN signaling indirectly promotes Dicer activity, thus generating more vsiRNAs.

While there are multiple independent experiments confirming the existence of antiviral vsiRNAs, others failed to detect vsiRNAs [106]. Many factors could contribute to the failures, including virus species, infection conditions/stages, cell types, RNA cloning strategies and analysis methods. It seems that vsiRNAs bear all the features of authentic siRNAs, including the ~22-nt size, phased distribution pattern with 2-nt 3′ overhangs, and more importantly, AGO2 association. Like other Dicer products including miRNAs and non-viral siRNAs, these AGO2-bound vsiRNAs also prefer small RNAs with 5′ U [98,99]. However, AGO2 association can not constitute the authenticity of antiviral RNAi since AGO2 could selectively bind 5′ U small RNAs generated by any pathway.

One of the core questions that remains unanswered in antiviral RNAi is whether vsiRNAs are functionally relevant in clearing viruses in differentiated cells in which IFN signaling is fully functional, playing predominant roles. A recent study by Han et al. found that vsiRNAs generated in differentiated mouse cells (mouse embryonic fibroblast cells or MEF) infected by NoV are indeed loaded to AGO2, forming functional RNA-induced silencing complexes (RISC), as demonstrated using in vitro slicer activity assays [17]. Surprisingly, NoV without VSR B2 was cleared from the mice lacking IFN signaling in a Dicer/AGO2-dependent manner (by RNAi). The paper found that B2, as a dsRNA binding protein, promotes virus infection primarily via inhibiting Dicer-dependent siRNA biogenesis and RISC activities. The mechanism of RISC inhibition was not explored. Interestingly, the lowered RISC activity was only limited to vsiRNAs but not to miRNAs.

In addition, Han et al. presented three other discoveries [17], which were different from previous reports. First, VSR B2 did not affect the induction of IFN signaling and RNase L mediated RNA degradation, while previous studies indicated that VSR may inhibit IFN signaling [10,102]. Second, IFN does not inhibit miRNA RISC activity, while a previous study proposed that in 293T cells, miRNA RISC activity was inhibited [107]. Third, the vsiRNA biogenesis was not inhibited by IFN signaling since Stat1/2^-/-^ mice (IFN signaling defective) exhibited similar levels of vsiRNAs as Rag1^-/-^ mice (intact IFN signaling). However, a previous study using artificial dsRNAs suggested that siRNA biogenesis was inhibited by IFN signaling [16,99]. In all, Han et al. propose that VSR primarily inhibits Dicer and RISC and does not affect IFN signaling.

This finding that mammalian cells are able to use RNAi to fully clear RNA viruses is exciting. Targeting VSRs to inhibit virus infection in mammalian cells may prove feasible. Even if this strategy may not eradicate viruses completely, it could slow down virus infection and alleviate clinical symptoms. The discoveries presented by Han et al. [17] will certainly spur more discussions and investigations in antiviral research.

## 7. Conclusions

Small RNAs play important antiviral roles, as exemplified by the CRISPR Nobel Prize this year and RNAi Nobel prize in 2006. Ideally, the small RNA based sensors are more specific and easy to design than conventional chemical and antibody drugs. However, viral RNAs are usually protected by proteins and may not be accessible. It is still challenging to design more target-specific and efficient strategies for delivering small RNA based drugs. Since many RNA viruses encode RNAi inhibitors, small molecule drugs targeting these inhibitors have potential in inhibiting virus infection. IAV strictly utilizes a WG (W represents A or U) motif to initiate mRNA transcription [5]. This motif can be used to develop nucleotide analog drugs, which may be more efficient and specific than the currently available drugs.

## Figures and Tables

**Table 1:** The roles of small RNAs in virus–host interaction.

Type	Roles Related to Viral Infection	Ref.
Capped small RNAs	Priming IAV mRNA synthesis. Serving as precursors for *C. elegans* piRNAs, which can be used as virus sensors. Serving as miRNA precursors.	[5,6,11,12,13,14]
siRNAs	Regulating gene expression. Guide host machinery to cleave viral RNAs.	[9,10,15,16,17]
Host miRNAs	Regulating gene expression. Required for viral RNA transcription.	[8,9,10]
Viral miRNAs	Regulating gene expression. Inhibiting host antivirus mechanisms.	[7,8,9]
piRNAs	Serving as virus sensors. Guide host machinery to cleave viral RNAs.	[18,19]
crRNAs	Serving as virus sensors. Guide host machinery to cleave viral RNAs.	[20,21]
snRNAs	Serving as cap donors for IAV mRNAs.	[6,12]
